# Magnitude and concurrence of anxiety and depression among attendees with multiple sclerosis at a tertiary care Hospital in Oman

**DOI:** 10.1186/s12883-015-0370-9

**Published:** 2015-08-05

**Authors:** Abdullah Al-Asmi, Salim Al-Rawahi, Zahir Saif Al-Moqbali, Yahya Al-Farsi, Musthafa M. Essa, May El-Bouri, Roopa P. Koshy, Arunodaya R. Gujjar, PC Jacob, Abeer Al-Hodar, Samir Al Adawi

**Affiliations:** Department of Medicine, College of Medicine and Health Sciences, Sultan Qaboos University, Muscat, Oman; Department of Behavioral Medicine, College of Medicine and Health Sciences, Sultan Qaboos University, P.O. Box 35, Al-Khoudh 123, Muscat, Sultanate of Oman; Department of Family Medicine and Public Health, Sultan Qaboos University, College of Medicine and Health Sciences, Muscat, Oman; Department of Food Science and Nutrition, College of Agricultural and Marine Sciences, Sultan Qaboos University, Muscat, Oman

**Keywords:** Multiple Sclerosis, Anxiety, Depression, Hospital Anxiety and Depression Scale, Expanded Disability Status Scale, Oman, Arab/Islamic

## Abstract

**Background:**

Anxiety, depression and functional impairments are commonly reported by persons with multiple sclerosis (PwMS) but no data, to our knowledge, has emerged from an Arab Islamic population. The study aims to investigate the prevalence of anxiety, depression and related disabilities among PwMS attending tertiary care in Sultan Qaboos University Hospital (SQUH), one of the urban hospitals in Oman.

**Methods:**

Consecutive and consenting PwMS (n = 57) and healthy subjects (n = 53) completed the following measures: *Hospital Anxiety and Depression Scale* (HADS) which was used to measure anxiety (cut-point >7) and depression (>7); and *Expanded Disability Status Scale* (EDSS) to measure the level of disability (≥5). Characteristics such as socio-demographic and clinical variables were also explored.

**Results:**

Fifty seven subjects with multiple sclerosis (MS) met the inclusion criteria. The majority of them were females who were 40 years old or younger and the majority were employed and unmarried. Approximately 86 % of the participants were using beta interferon, 96 % scored ≥5 in EDSS. MS of the Relapsing-Remitting type constituted the majority of the cohort (94 %). Approximately 35 % and 51 % endorsed symptoms of anxiety and depression respectively. The MS group scored significantly higher than controls on HADS measurements of depression and anxiety.

**Conclusion:**

Disability and symptoms of anxiety and depression are common among the PwMS attendees of tertiary care hospital in Oman. Such psychosocial variables have been largely unreported emerging from non-western populations. As these variables are strong indicators of the burden of MS, resolute effort is needed to address such psychosocial dysfunctions in the algorithms of care for PwMS in the Arab Islamic part of the world.

## Background

Multiple Sclerosis (MS) is an inflammatory condition that tends to have a detrimental effect on cognitive, emotional and social functioning [1]. It has been estimated that 23.4 per 100,000 people in different populations around the world are likely to fulfill varying spectrums of MS [2, 3]. However, there is a discrepancy in rates of MS, with higher rates being reported among Caucasian populations and the lowest incidences in sub-Saharan Africans [4].

According to the Atlas of MS compiled by the Multiple Sclerosis International Federation and the World Health Organization [5], the Arabian Gulf, which is geographically defined as part of the Western Asia region, is a low-risk zone for MS. This low-risk status remains despite the recent affluence and rising tide of ‘diseases of affluence’ [6] including MS [7–9] in the region. However, there are emerging epidemiological surveys which are challenging this low risk hypothesis. Benamer et al. [10] have reviewed studies reporting on the rate of MS from Arab populations. Nineteen articles have been found from the review covering publications ranging from 1975 to 2007. The prevalence rate varied from 4 to 42 per 100,000 people. More recently, the prevalence and incidence of MS was reported at 85.05 and 6.88 per 100,000 persons in Kuwait respectively [11]. While the magnitude of those affected by MS has been previously explored, little has been documented on the psychological functioning of persons with multiple sclerosis (PwMS) in the Arabian Gulf regions.

Some studies in the Arabian Gulf regions indicate clinically heterogeneous diagnoses of MS [12]. Akhtar et al. [13] have examined correlations of MS among newly diagnosed individuals in Qatar. The study indicates that MS in that particular population is marked by milder clinical symptoms that tend to be of the ‘relapsing remitting’ type, though with “aggressive radiologic disease presentation”. The study in Oman reports a high incidence of optic-spinal disease and a negligible rate of oligoclonal bands [14]. In this context, the clinical pattern in Oman resembles Asian populations [14] while the pattern in Qatar and Iraq appears to be similar to what is observed in ‘Western’ populations [13, 15]. It is not clear how far-reaching these different clinical patterns are in terms of their effect on psychological functioning. However, a stressful life event has been suggested to exacerbate symptoms of MS [16].

Although MS is a debilitating neurological condition which tends to trigger psychological symptoms such as anxiety and depression, no data indicating this has been searched among PwMS in Arab populations. Depressive symptoms have been reported to occur in figures that range from 15 % to above 50 % of PwMS [17–22]. In addition to depression, variant forms of anxiety disorders have been reported to be common among PwMS [23–25]. Korostil and Feinstein [26] examine the presence of anxiety among 140 consecutive clinic attendees using the Structured Clinical Interview, as well as the *Hospital Anxiety and Depression* (HADS) ‘symptoms checklist’. The study indicates that approximately 36 % of the participants were marked with an anxiety disorder, while approximately 19 % had symptoms of generalized anxiety disorder. Both depression and anxiety are associated with impaired social functioning, lower quality of life and greater utilization of healthcare services which in turn increases the cost of caring for such a population [27]. Therefore, establishing a mechanism for the early diagnosis and intervention of these disorders in PwMS would be essential. An extensive search on the available literature reveals that there is a dearth of studies on anxiety and depression among PwMS in Arabian Gulf. The present study has embarked with interrelated aims. The first aim is to audit whether consensus emerging elsewhere that mood disorders such as depression [21] and anxiety [28, 29] are common in PwMS holds true for non-western populations. The specific aim is to explore the rate of anxiety and depression in a consecutive sample population of PwMS seeking consultation at a teaching hospital in Oman. The second aim is to explore the relationship between the level of mood score on the HADS symptom checklist with clinical variables and socio-demographic background. An understanding of psychosocial variables is important because they have implications on the outcome of the illness.

## Methods

### Subjects

A cross-sectional study was conducted during the period from January 01, 2012 to December 31, 2013, wherein MS cases were recruited, then compared to subjects negative for MS. The study used a consecutive sampling plan procedure, wherein every patient with stable symptoms of MS who came for routine consultation at the neurology outpatient clinic at the Sultan Qaboos University Hospital (SQUH) were enrolled.

SQUH serves an ethnically diverse community located in the coterminous Muscat governance. Being the only teaching hospital in Oman and as one of the two referral centers for neurology in the country, it caters to referrals from all regions of the country. The annual patient-visit volume per year for SQUH is in excess of 100,000.

The inclusion criteria constituted those who consented to participate in this study who have fulfilled MS nomenclature as depicted in McDonald Revised criteria [30].

Exclusion criteria included those who are suspected to have concurrent brain disorders, persistent and pervasive psychiatric conditions or cognitive impairment or dementia that would render them incapable of participating meaningfully in the study. Cognitive impairment was operationalized as those who scored less than <18 points in the *Mini–Mental State Examination* (MMSE) or Folstein Test [31]. Similarly, participants with current or recent (i.e. within the previous 30 days) MS exacerbations were excluded for rationale that has been outlined elsewhere [32]. Neurologists who are experienced in diagnosing and treating MS conducted all the required neurological evaluations as well as coding each participant in terms of fulfilling the McDonald Revised criteria [33]. For the present purpose, the subtypes of MS were defined as ‘relapsing remitting’, ‘secondary progressive’ and ‘primary progressive’ [33]. Senior medical students who were unaware of the participants’ clinical features administered HADS to the consenting participants. Every person who presented to the clinic with a diagnosis of MS was requested to participate, and if consented, were screened and officially diagnosed using the McDonald Criteria. The medical students, also unaware of the patients’ clinical features, administered the MSSE and HADS tests immediately after the neurology appointment. This meant that all psychological measures were administered in one visit.

Participants in the healthy control sample were recruited volunteers from among either relatives of the client seeking consultation or consenting staff from the College of Medicine at Sultan Qaboos University. To qualify as a control sample, the participant was required to confirm to the researcher that as to his/her knowledge, she/he is clear of any health conditions relating to MS.

A total of 126 participants had the potential to be included in the study, where 63 were MS cases and the rest, controls. Out of the total cases, 57 met the inclusion criteria and consented. Two of the MS cases were excluded because they did not completely fulfill the McDonald diagnostic criteria, while 3 others refused to participate. Out of a total of 63 eligible control subjects, 4 refused to participate, and 6 were excluded due to these reasons: 2 were suspected to have neurological symptoms, and 4 had incomplete/missing information.

The study was approved by the Medical Research Committee and the Ethical Committee (MREC#464) of the College of Medicine and Health Sciences, Sultan Qaboos University. Participants were asked to sign a written consent.

### Measurement

#### Hospital Anxiety and Depression Scale (HADS)

Consenting participants were subjected to neurological examination and administered the Arabic-version of HADS. Psychometric properties of HADS have been established in Arabic speaking populations including Oman [34, 35]. HADS, which is portable and easy-to-use, measures a person’s current anxiety and depression levels. Each subscale score ranges from 0 to 21, with higher scores representing poorer emotional well-being. A previous study of the Oman’s population had identified a cut-off point of 8/21 for anxiety or depression. In a previous study [32], two cut-off scores (i.e., 8/21 and 11/21) were explored for each subscale. For theoretical reasons, the score of the present participants in the two cut-off point (8/21 and 11/21) were also explored.

HADS, as specifically designed for clients with general psychiatric conditions, is sensitive to the probable caseness for anxiety or depression in clinical populations characterized with ‘potential somatic confounders’ [32] including MS [36, 37].

#### Degree of disability

Degree of patient disability was also sought using criteria defined by the *Kurtzke Expanded Disability Status Scale* (EDSS) [38]. The EDSS solicits the presence of disability in eight Functional Systems (FS) which, in turn, are expressed as a Functional System Score (FSS) in each of these. The protocol for quantifying disability in PwMS has been described in detailed elsewhere [39]. In brief, the progenitor of this measure has operationalized FS to stem from the integrity of pyramidal, cerebellar, brainstem, sensory, bowel and bladder, visual, cerebral and other bodily systems. EDSS categorizes the severity of disability ranging from 1 to 10 with a higher score denoting a higher index of severity.

In addition to HADS and EDSS, socio-demographic information as well relevant clinical information was sought from the referral note, medical records or during the consultation. Table 1 depicts clinical and socio-demographic information that was gathered for this study.

**Table 1:** Socio-demographic and clinical characteristics of persons with multiple sclerosis (PwMS) versus healthy subjects attending a tertiary hospital, Oman

Characteristics	Total (N = 110)	PwMS (N = 57)	Healthy subjects (N = 53)	P value
	N (%)	N (%)	N (%)	
Gender				0.04
Female	63 (57.3)	41 (71.9)	22 (41.5)	
Male	47 (42.7)	16 (28.1)	31 (58.5)	
Age				0.05
40 or less	91 (82.7)	51 (89.5)	40 (75.5)	
More than 40	19 (17.3)	6 (10.5)	13 (24.5)	
Education				0.99
12 years or less	54 (49.1)	28 (49.1)	26 (49.1)	
More than 12 years	56 (50.9)	29 (50.9)	27 (50.9)	
Marital status				
Unmarried	64 (58.2)	35 (61.4)	29 (54.7)	
Married	46 (41.8)	22 (38.6)	24 (45.3)	0.04
Employment				
Employed	51 (46.3)	7 (12.3)	44 (83.1)	0.02
Unemployed	59 (53.7)	50 (87.7)	9 (16.9)	
Interferon				
Yes		49 (86)		
No		8 (14)		
Expanded Disability Status Scale				
≤5		52 (96.3)		
>5		2 (3.7)		
Course of Disease				
Relapsing Remitting (RR)		54 (94.7)		
Secondary Progressive (SR)		1 (1.8)		
Primary Progressive (PR)		2 (3.5)		

#### Data analysis

Chi-square analyses were used to evaluate the statistical significance of differences among proportions of categorical data. The non-parametric Fisher’s exact test (two-tailed) replaced the Chi-square test in cases of small sample size, where the expected frequency was less than 5 in any of the cells in 2 x 2 tables. The Independent Student's t-test and Analysis of Variance (ANOVA) were used to evaluate the statistical significance of differences in between means of continuous data. The non-parametric Wilcoxon Rank test was used to explore differences of means of continuous variables as is used in cases of small-sized data. The depression prevalence estimates were calculated by dividing the total number of cases with depression by the total number of the index population.

The binomial distribution method was used to calculate the 95 % confidence intervals (CI) of the rate estimates using the Wald method. The odds ratios (OR) and 95 % confidence intervals (CI) obtained from logistic regression models were taken as the measures of association between depression and selected socio-demographic predictors. The following variables were adjusted for in the regression modeling: gender, education, age, EDSS score, interferon use, disease duration. All statistical analyses were performed using the Statistical Package for Social Sciences (SPSS) software (Version 20.0, IBM, Chicago, Illinois, USA). A significant association is considered if the 95 % CI does not include the value 1.0; and a cutoff *p*-value of less than 0.05 is used for all tests of statistical significance in this study.

## Results

Fifty seven subjects with MS met the inclusion criteria, and were compared to 53 controls. Table 1 shows the socio-demographic characteristics of multiple sclerosis (MS) group versus those of the control group. Overall, the MS group tends to include more unmarried females who are 40 years old or younger. Proportionately, the MS group tended to be employed and unmarried. The level of education was comparable among the two groups.

Table 2 shows the estimates of prevalence of the symptoms of anxiety and depression among the cases and control groups. Among the 57 MS cases, 29 (50.8 %) were found to suffer from symptoms of anxiety and 20 (35.1 %) to suffer from symptoms of depression; while among the control group, the proportions of those with symptoms of anxiety and depression were 35.1 % and 18.9 %, respectively. Both proportions of the symptoms anxiety and depression were significantly higher among cases compared to control group (P ≤ 0.05).

**Table 2:** Estimated prevalence (95 % CI) of anxiety and depression among persons with multiple sclerosis (PwMS) and Healthy Subjects attending a tertiary hospital, Oman

Outcome	Case (N = 57)		Healthy subjects (N = 53)		P value
	*N (%)*	P* (95 % CI)	*N (%)*	P* (95 % CI)	
Anxiety	29 (50.8)	50.8 (38.3, 63.4)	14 (26.4)	26.4 (16.3, 39.7)	0.01
Depression	20 (35.1)	35.1 (24.0, 48.1)	10 (18.9)	18.9 (10.4, 31.6)	0.05

*P: Prevalence estimate is per 100 patients

Table 3 shows adjusted odds ratio estimates for the associations between MS and symptoms of anxiety and depression. The adjusted ORs generated by logistic regression models indicated that multiple sclerosis was associated with substantial risks of both symptoms of anxiety (OR = 2.43; 95 % CI 1.13, 6.82; P = 0.03) and depression (OR = 2.11; 95 % CI 0.92, 6.72; P = 0.07).

**Table 3:** Adjusted odds ratios (95 % CI) of associations between multiple sclerosis and each of anxiety and depression as quantified by the Hospital Anxiety and Depression Scale among attendees of a tertiary hospital in Oman

Variable	OR^a^	95 % CI	*P* value
Anxiety	2.43	1.13, 6.82	0.03
Depression	2.11	0.92, 6.72	0.07

^a^Adjusted odds ratio estimates were generated by considering the confounding effects of age, gender, marital status, and employment status

Table 4 compares the anxiety and depression scores according to HADS in between MS case versus control groups. Overall, the HADS scores on anxiety among MS patients were significantly higher (8.3 vs. 4.9) than that among the control group (P = 0.04). A similar pattern was also observed along the HADS scores on depression (6.5 vs. 4.1; P = 0.05). This pattern of observing higher scores among MS cases compared to controls was also persistent upon further stratifying HADS scores according to proposed cutoffs (i.e. 8/21) which identified pathological HADS scores.

**Table 4:** Performance of anxiety and depression sub-scale of Hospital Anxiety and Depression Scale (HADS) among persons with multiple sclerosis (PwMS) versus Healthy subjects attending a tertiary hospital in Oman

Characteristics	PwMS (N = 57)	Healthy subjects (N = 53)	P value
	N (%) or mean (SD)	N (%) or mean (SD)	
HADS (Anxiety)			
Mean (SD)	8.3 (4.8)	4.9 (4.7)	0.04
Group- n (%)			
≥11	22 (38.6)	9 (17.0)	0.07
≥8	31 (54.4)	15 (28.3)	0.04
HADS (Depression)			
Mean (SD)	6.5 (5.1)	4.1 (4.4)	0.05
Group- n (%)			
≥11	12 (21.1)	7 (13.2)	0.08
≥8	22 (38.6)	12 (22.6)	0.06

Table 5 shows the associations between HADS scores for anxiety and depression and selected socio-demographic and clinical characteristics. On average, higher scores of anxiety on HADS were reported among participants who were female, married, employed, and with more than 12 years of education. Clinically, higher scores of HADS on anxiety were associated with no intake of interferon, EDSS greater than 5, and secondary progressive MS. Higher scores of HADS on depression were reported among participants with similar socio-demographic and clinical characteristics to those observed with HADS on anxiety, except that they were higher among participants who were unmarried and with less than 12 years of education.

**Table 5:** Factors associated with each subscales of Hospital Anxiety and Depression Scale depression (HADS) among of persons with multiple sclerosis (n = 57 attending a tertiary hospital in Oman

	HADS Anxiety		HADS Depression	
	Mean (SD)	P value	Mean (SD)	P value
Sex		0.03		0.27
Female	7.3 (5.0)		5.7 (4.6)	
Male	5.3 (4.9)		4.8 (4.6)	
Educational level		0.02		0.12
≤12 years	6.1 (5.1)		5.6 (4.8)	
>12 years	6.8 (4.9)		5.1 (4.3)	
Marital status		0.12		0.08
Unmarried	6.3 (5.2)		5.8 (4.5)	
Married	6.8 (4.8)		5.2 (4.6)	
Employment		0.01		0.32
Employed	8.2 (4.8)		6.2 (4.2)	
Unemployed	5.8 (4.9)		5.3 (4.9)	
Interferon		0.24		0.09
Yes	7.9 (4.8)		6.3 (4.4)	
No	10.1 (4.7)		9.2 (4.1)	
EDSS		0.06		0.21
≤5	8.1 (4.7)		6.5 (4.3)	
>5	14.5 (3.7)		10.5 (3.5)	
Course of disease		0.13		0.26
Relapsing remitting (RR)	8.1 (4.7)		6.7 (4.4)	
Secondary progressive (SP)	14.0		11.5	
Primary progressive (PP)	3.0 (1.2)		4.0 (1.1)	

Table 6 shows the associations between probable for depression and selected factors as obtained from multi-variate logistic regression. Overall, a slight increase in risk of pathological depression was observed with advanced age, female gender, and less education. A more substantial increase in risk of pathological depression was observed with increased duration of disease (OR = 2.32; 95 % CI 0.52, 7.53) and EDSS >5 (OR = 5.83; 95 % CI 0.26, 9.4). Nonetheless, all obtained associations were not statistically significant (P-value >0.05).

**Table 6:** Factors associated with HADS depression scores (cut-off 8/21) among of persons with multiple sclerosis (n = 57) attending a tertiary hospital in Oman

Variable		Odds ratio (95 % CI)			
	Mean (SD)	Crude	P-value	Adjusted	P-value
Age > 40 years	5.6 (4.1)	1.58 (0.48, 5.23)	0.45	1.28 (0.13, 7.33)	0.83
Female gender	5.7 (4.6)	1.32 (0.48, 3.63)	0.58	1.14 (0.24, 5.32)	0.87
Education ≤ 12 years	5.6 (4.8)	1.05 (0.63, 1.76)	0.85	1.13 (0.57, 2.26)	0.71
Disease duration ≥ 5 years	6.9 (4.5)	1.46 (0.40, 5.31)	0.56	2.32 (0.52, 7.53)	0.28
No use of interferon	9.2 (4.1)	3.28 (0.21, 6.8)	0.29	3.91 (0.23, 7.9)	0.24
EDSS score > 5	10.5 (3.5)	4.20 (0.24, 7.3)	0.32	5.83 (0.26, 9.4)	0.27

## Discussion

The first aim of this study was to examine whether anxiety and depressive symptoms that are common in PwMS in Euro-American populations [21, 37, 40] are also present in cases in Oman. To our knowledge, there is a dearth of studies examining the presence of psychological distress in PwMS in Arabian Gulf countries, and Oman is no exception. Previous studies have shown that anxiety and depressive symptoms are common in various clinical populations in Oman [34, 35].

The present study indicates that 50.8 % of the participants endorse themselves as having depressive symptoms, according to the cut-off point of HADS. Studies from other populations have indicated that 29-54 % of PwMS tend to exhibit various spectrums of depressive illness [17, 41]. Many factors could determine the wide variations in the rate of depressive symptoms, including the different types of instruments employed to quantify the presence of depression as well as the heterogeneous cohorts employed. It is worthwhile to note the rate of depressive symptoms in the present study falls in the range of earlier studies that have utilized HADS [42].

This study indicates that 35 % of PwMS manifest anxiety symptoms. These results are similar to a study reported by Smith and Young in the United Kingdom [43], where they found that among 88 consecutive PwMS, 34 % scored in the clinical range in the anxiety subscale of HADS.

The present observation that the rate of depressive and anxiety symptoms as endorsed by the HADS scale in PwMS in tertiary care in Oman is common raises an interesting discourse. According to some commentators (e.g. Dwairy [44]), Arab societies tend to lean towards a ‘collective’ social orientation which is contrasted to individualistic social orientation commonly found in Western countries. In order to preserve social harmony, such a society tends to discourage the expression of emotion and therefore relegates emotional distress. Instead, distress is expressed in somatic terms, a feat that is orthogonal to what may constitute emotional distress in the psychiatric nomenclature such as *Diagnostic and Statistical Manual of Mental Disorders* (DSM) [45] and *the International Statistical Classification of Diseases and Related Health Problems* (ICD-10) [46]. Within such a context, an examination of anxiety and depressive symptoms in non-Western societies is hampered by the fact that symptoms are articulated differently. Rather than simply relying on the established cut-off point for HADS to identify the presence of anxiety and depressive symptoms in PwMS in Oman, the present study uses ecological validity by comparing the performance of HADS with healthy participants. Within such a comparison, the present data unequivocally suggests that PwMS have higher scores in anxiety and depressive symptoms compared to healthy controls. The present study challenges the assumption that people in non-Western societies are devoid of emotional distress.

There is a debate on the ‘origin’ of anxiety and depressive symptoms in PwMS. One hypothesis suggests that pathophysiological processes that are an integral part of MS tend to trigger anxiety and depressive symptoms [47, 48]. Functional neuroimaging data have suggested that attenuation of the activities in frontal and temporal lobes of the right hemisphere does not only correlate with presence of MS but also the occurrence of anxiety and depressive symptoms [49, 50]. The alternative hypothesis is that anxiety and depressive symptoms in MS are a reaction to living with MS. Regardless of whether it is a reaction or an integral part of MS, the much ignored psychological need of those PwMS should be considered in the algorithm of care for the disease. Along with establishing psychological services, studies are needed to shed light on how anxiety and depressive symptoms among PwMS are perceived in Oman’s society.

The second aim of this study was to explore the relationship between anxiety and depressive symptoms with clinical and socio-demographic variables. The participants who scored highly for both anxiety and depressive symptoms appear to have similar clinical characteristics, for example, having been treated with interferon, having a high level of disability as quantified by an EDSS (score > 5) and fulfilling the diagnosis for ‘Secondary Progressive’ type MS. Other correlations are worth highlighting. HADS scores on both anxiety and depression were found to be markedly higher in females, a feat that is congruent with the international trend [51]. The present study indicates that higher scores in indices of anxiety are more common among married females, in particular, amongst those who have achieved more than 12 years of education. In emerging economies such as the one found in Oman, women are often required to juggle between career and family life [52] and all the stress and anxiety it may entail. In contrast, a depressive symptom was found to be higher in unmarried females with a lower educational attainment. In traditional Omani society, it is stated that ‘a wedding is a blessed garment’ which, in turn, implicitly stigmatizes unmarried women [53]. It is also worthwhile to note that this study found that the majority of participants were unmarried. On the whole, the view that there is a preponderance of unmarried people among PwMS has also been reported in other populations [54]. It is possible that sexual dysfunction is common in PwMS [55, 56] and the disability it entails may render these women as ineligible for marriage in Omani society.

The study should be viewed as a sentinel project and therefore some obvious limitations need to be highlighted. *Firstly*, the cohort was a convenient sample and could be a biased sample of self-selective people who were referred to tertiary care in an urban area. Such biased sample and the nature of catchment area hamper the generalization of this study. *Secondly,* although HADS appeared to be valid for use in the Omani population [34, 57], the HADS test has not been standardized for Arabic speaking PwMS. Similarly, HADS is marred with limitations when compared to a semi-structured interview since, for example, it is known to solicit the presence of ‘hedonic tone’ rather than other characteristics of depression such as hopelessness, guilt and low self-esteem [57].

The presently observed rate of anxiety and depressive symptoms present potential ‘case-ness’ since the ‘gold standard interview’ was not employed. It is worthwhile to note that HADS provides a quick screen, not a diagnosis. The present observed rate appears to echo international standards even to those studies that have utilized the gold standard interview. The *third* limitation of this study stems from the conspicuous absence of measures that solicit the presence of fatigue. There is ample evidence suggesting that fatigue tends to influence psychological state and vice-versa [58] and many available scales are ‘contaminated’ by the MS symptoms such as tiredness and impaired cognition. This is likely to dent the reliability of the instrument in eliciting the symptoms of anxiety or depression [59]. Future studies should examine whether depression and fatigue are ‘twin sisters’ with overlapping or orthogonal symptoms [60]. Additionally, this study would have had higher heuristic value if the participant’s duration of symptoms were established. Future studies could address these limitations. On the whole, larger studies possibly with age and gender matched controls, with formal confirmation of emotional changes of those patients with positive screening scores, stratification of patients based for example on disease duration, etc. may provide more robust information regarding the prevalence and possibly the risk factors for depression and anxiety in MS.

## Conclusion

To our knowledge, this is the first study to explore psychosocial variables in PwMS in an Arab/Islamic population. The findings is congruent with international trends in terms of the magnitude of anxiety and depressive symptoms. Similarly, various correlates of anxiety and depressive symptoms observed in the present study appear to echo the international trend with slight variation due to socio-cultural factors. The present findings have various implications. A larger study with more robust methodology is needed to scrutinize the present findings so that a concerted effort can be contemplated to improve the psychosocial services for PwMS, including detection and culturally sensitive psychological interventions. In many non-Western cultures, there is a tendency to neglect these services for clients with chronic illnesses. This study suggests that psychological disorders are common in PwMS. Therefore, development of services to attend to their psychological disorders is warranted.
